# Are you my mother? When host genetics and gut microbiota tell different phylogenetic stories in the Africanized honey bee hybrid (*Apis mellifera scutellata* × sspp.)

**DOI:** 10.1128/spectrum.02475-24

**Published:** 2025-05-28

**Authors:** Kilmer Oliveira Soares, Celso José Bruno de Oliveira, Luis Eduardo Martínez Villegas, Priscylla Carvalho Vasconcelos, Adriana Evangelista Rodrigues, Christopher Madden, Vanessa L. Hale

**Affiliations:** 1 Department of Animal Science, College of Agricultural Sciences, Federal University of Paraiba, Areia, Paraíba, Brazil; 2 Global One Health Initiative (GOHi), Ohio State University 2647, Columbus, Ohio, USA; 3 College of Food, Agricultural, and Environmental Sciences, The Ohio State University 2647, Columbus, Ohio, USA; 4 Department of Veterinary Preventive Medicine, College of Veterinary Medicine, The Ohio State University 70728, Columbus, Ohio, USA; 5 Center of Microbiome Science, The Ohio State University 2647, Columbus, Ohio, USA; China Agricultural University, Beijing, China

**Keywords:** Brazil, Africanized honey bee, gut microbiota, honey bee, European honey bee, Afriacn honey bee, evolution, *Apis mellifera*

## Abstract

Africanized honey bees (*Apis mellifera scutellata* × sspp.) originated in Brazil through the crossbreeding of African (*A. mellifera scutellata*) and European (*A. mellifera* sspp.) honey bee subspecies. African genes came to dominate in these hybrid honey bees over time. Gut microbiota co-evolve with their hosts and generally reflect host phylogeny. To examine if this was true in Africanized honey bee hybrids (also known as *scutellata*-European hybrids), we compared the gut microbiota (16S rRNA) of three honey bee subspecies: African, European, and Africanized bees. Publicly available sequencing data from five honey bee studies were downloaded from the National Center for Biotechnology Information (NCBI). European bee samples (*n* = 42) came from the United Kingdom, Switzerland, and the United States. African bee samples (*n* = 82) came from Kenya. Africanized bee samples (*n* = 10) came from Brazil. Unexpectedly, Africanized honey bee gut microbiota was far more similar to European bees than to African bees despite the closer host genetic relationship between African and Africanized bees. All three subspecies shared similar relative abundances of core taxa. We posit that the similarity in gut microbiota between Africanized and European honey bees arose from the nature of the crossbreeding and the social/environmental transmission of gut microbiota within hives. Namely, African queens took over European hives. However, the hybrid offspring acquired their gut microbiota from European nurse bees and European hive materials, resulting in the stable transmission of European gut microbiota across generations. Our results provide an intriguing insight into the potential ecological, social, and environmental factors that shape the gut microbiota of the Africanized honey bee hybrid.

**IMPORTANCE:**

Africanized honey bee hybrids originated in Brazil through the crossbreeding of African and European honey bee subspecies. In this study, we examined the gut microbiota of all three honey bee subspecies (African, European, Africanized). A few core microbiota were shared across all subspecies. Interestingly, while African honey bee genes dominated in the Africanized honey bee hybrids, their gut microbial composition was most similar to European bees. This is likely related to the way these bees were crossbred—with African queens taking over European hives, while gut microbial inoculation of hybrids came from European nurse bees and European hive materials. Gut microbiota are critical to honey bee health, and studying the gut microbiota of closely related honey bee subspecies helps understand the factors that influence gut microbial composition. This is important for our broader understanding of honey bee health, conservation, and evolution.

## INTRODUCTION

**Fig 1:**
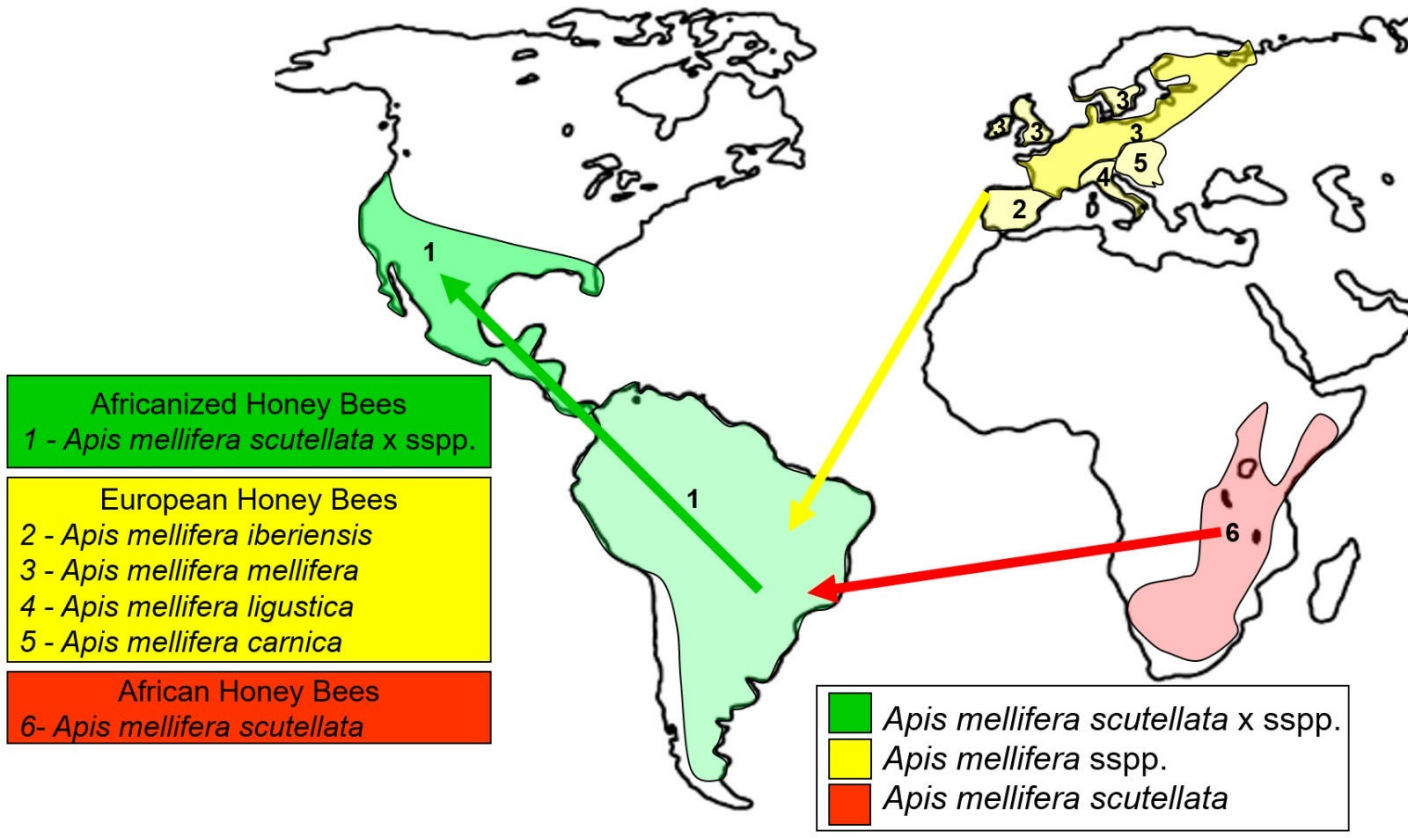
Honey bee introductions, hybridization, and expansion. Yellow route: European honey bees (*Apis mellifera* sspp.) were introduced in Brazil between 1839 and 1880. Red route: African honey bee queens (*Apis mellifera scutellata*) were introduced in Brazil in 1956 (9). Green route: natural expansion of Africanized honey bees (*Apis mellifera scutellata* × sspp.) across Americas (10).

Honey bees have spread throughout the world via migration, hybridization, and human importation (1–4). Thirty-three genetically distinct *Apis mellifera* honey bee subspecies have been identified and are geographically distributed across three main regions: Africa (11 subspecies), Western Asia and the Middle East (nine subspecies), and Europe (13 subspecies) (5). Brazil is home to a hybrid honey bee subspecies (*Apis mellifera scutellata* × sspp.) derived from a cross between African and European subspecies. European honey bees (*Apis mellifera* sspp.) were first introduced to Brazil in 1839 by priest Antonio Pinto Carneiro who imported bees from Portugal and Spain (6). Later, between 1845 and 1880, German and Italian immigrants introduced other European subspecies (*A. mellifera* sspp.) to Brazil (6). In 1956, Professor Warwick Estevam Kerr brought 26 African honey bee queens (*Apis mellifera scutellata*) from Tanzania and South Africa to Brazil. These queens escaped from their apiary and replaced the queens in the European bee colonies already established in Brazil. The African queens then mated with European drones, ultimately establishing a new hybrid subspecies (7). This was the origin of the Africanized (*Apis mellifera scutellata* × sspp.), also known as *scutellata*-European hybrids (3, 8) (Fig. 1).

**Fig 2:**
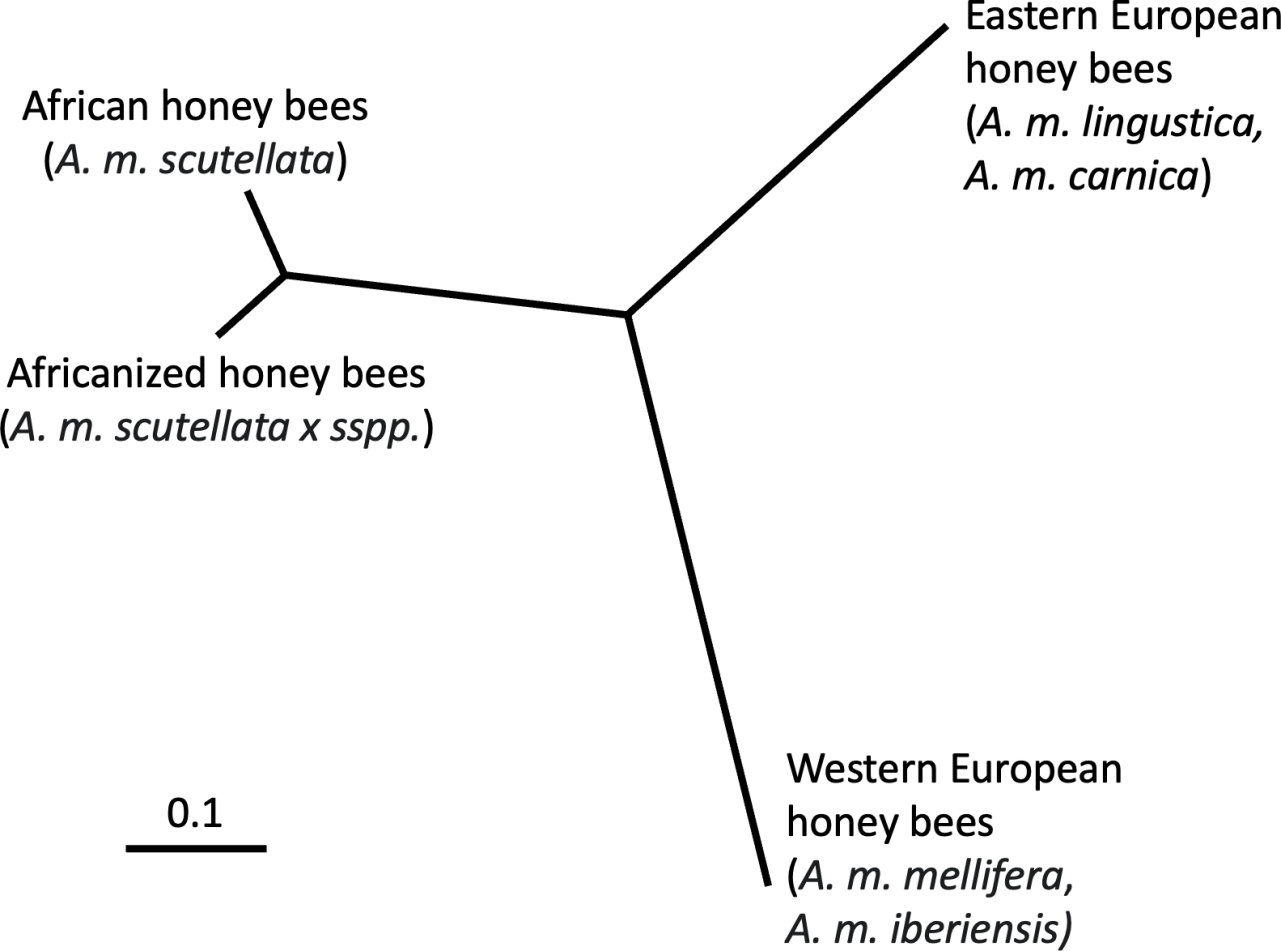
Genetic distance between African, European, and Africanized honey bee populations. Distances based on 95 single-nucleotide polymorphisms displayed in an unrooted UPGMA tree. Adapted from reference 13.

Honey bee subspecies exhibit differing biological characteristics (e.g., pathogen resistance, heat tolerance, behavior, resource use) that have supported their adaptation and expansion across varying environments, climates, and ecological pressures around the globe. African honey bee subspecies, for example, exhibit rapid colony growth, increased resistance to Varroa mites (11) and pathogenic viruses, and improved survival in neotropical environments as compared to European honey bee subspecies (4, 12). These same traits proved beneficial in Brazil, resulting in the dominance of African-bee derived genes (50–90%) in the Africanized honey bee hybrid and facilitating the spread of this hybrid subspecies (*A. mellifera scutellata* × sspp.) across the Americas (Fig. 1 and 2) (3, 4, 13–16). Although many phenotypic and genotypic differences have been characterized between African, European, and Africanized honey bees, one biological characteristic that has not been well explored in relation to honey bee phylogeny and hybridization is the gut microbiota.

Importantly, gut microbiota are critical to host health and the adaptation of hosts to new environments (17, 18). In fact, previous studies have already determined that honey bee gut microbes play a key role in nutrient degradation (19), bacterial and fungal pathogen defense (20–22), pesticide tolerance (23), and behavior (24, 25). Numerous prior studies have also demonstrated links between host phylogeny and the gut microbiome (26–29). Understanding the relationship between host phylogeny, hybridization, and the gut microbiota of these three honey bee subspecies (African, European, Africanized) is of significant importance, as it can provide insights into mechanisms of adaptation and evolution in honey bees. In this study, we compared gut microbiota of African, European, and Africanized honey bee subspecies. We hypothesized that Africanized honey bee gut microbiota (*A. mellifera scutellata* × sspp.) would represent a hybrid between African and European bee gut microbiota, and that Africanized bee gut microbiota would align with host phylogeny and be more closely related to African bee gut microbiota.

## MATERIALS AND METHODS

### Study design

**Fig 3:**
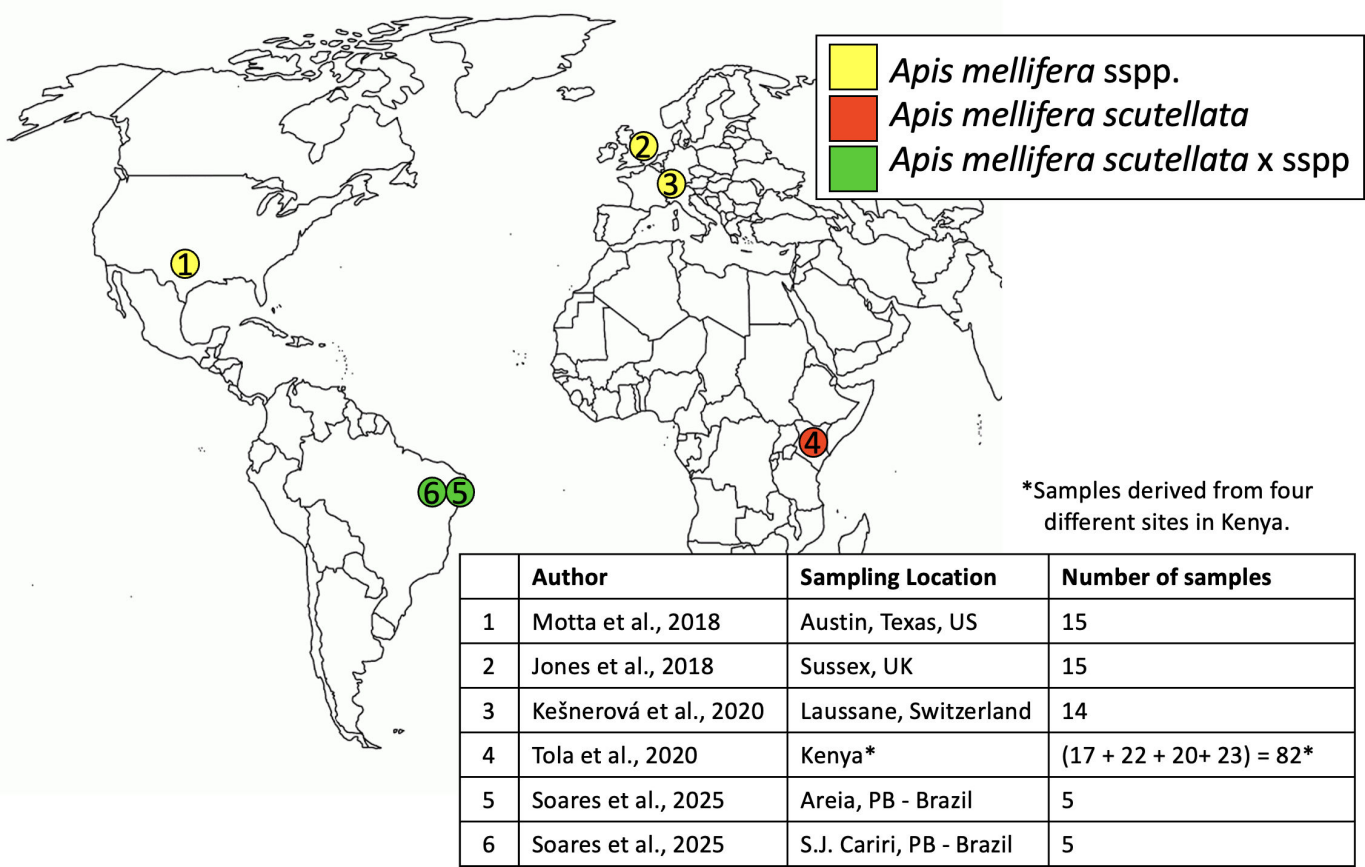
Sampling locations for European, African, and Africanized honey bees.

This study included publicly available 16S rRNA data from five previously published studies on honey bees (Fig. 3; Table S1). Data were downloaded from NCBI’s Short Read Archive or Zenodo. Sequencing platforms, primers, and extraction methods used in each study are listed in Table S1. All studies sampled free-ranging adult worker bees under natural field conditions. We exclusively used “control bee” samples if the study involved treatment and control groups. European honey bee samples came from Austin, Texas, United States (*n* = 15) (22), Sussex, United Kingdom (*n* = 15) (30), and Lausanne, Switzerland (*n* = 14) (18). Texas bees were confirmed as European honey bees based on a personal communication with N. Moran. African honey bee samples came from four sites in Kenya: Kakamega (*n* = 17), Kilifi Coast (*n* = 22), Kwale Coast (*n* = 20), and Nairobi (*n* = 23) (31). Africanized honey bee samples came from two municipalities of the State of Paraíba, Brazil: Areia (*n* = 5) and São João do Cariri (*n* = 5) (32).

### Sequence data processing and statistical analysis

Raw paired-end sequences were downloaded for each study separately and then merged using the command “qiime feature-table merge.” Sequences were then demultiplexed, filtered, denoised, and truncated to a length of 248 base pairs using QIIME 2-2020.2 (Bolyen et al., 2019) (see Appendix 1 for QIIME scripts). According to Liu et al. (33), V3–V4 amplicon analysis is compatible with V4 amplicon analysis after trimming to the same region (Appendix 1—Script 1). Chimeric sequences were removed, and DADA2 was used to parse sequences into amplicon sequence variants (ASVs) (34). Sequences were then aligned using “qiime fragment-insertion sepp” for phylogenetic analysis (35). Taxonomic composition of the samples was determined with a pretrained naive Bayes classifier with a 99% sequence similarity threshold for V3–V4 reference sequences (SILVA-132-99-nb-classifier.qza) and the “qiime feature-classifier classify-sklearn.” Reads identified as chloroplasts, mitochondria, unassigned, and eukaryota were removed from all samples.

Microbial composition and diversity were analyzed in QIIME 2-2020.2 (Bolyen et al., 2019). Microbial compositions (beta diversity) were compared between honey bee subspecies (European, African, Africanized) using permutational multivariate analysis of variance (PERMANOVA) based on Bray-Curtis, Jaccard, and weighted and unweighted Unifrac distances (36). Microbial communities were visualized using principal coordinate analysis and the Emperor plugin 2020.2.0 (37). Microbial diversity (alpha diversity) was assessed using observed ASVs, the Shannon diversity index (richness and abundance), Faith’s PD (phylogenetic diversity), and Pielou’s (evenness) diversity index (38). We assessed for normality using Shapiro-Wilk tests; then, as appropriate, used ANOVAs or Tukey’s or Kruskal-Wallis test at 5% probability to compare microbial diversities between groups using R version 4.1.0 (39).

Differentially abundant taxa were identified using an analysis of composition of microbiomes (ANCOM) (Mandal et al., 2015). ANCOM is a linear regression model that compares log ratios of features (taxa) between groups to identify taxa that are significantly more abundant in one group than another. We also identified core taxa defined here as taxa present in 90% of the samples using the command “qiime feature-table core-features.” Both analyses were performed at the genus level. The relative abundances of core microbes were compared by honey bee subspecies using a one-way ANOVA, followed by a pairwise Kruskal-Wallis rank sum test (Appendix 1—Script 2). To assess the strength (specificity/sensitivity) of the relationship between specific taxa and honey bee subspecies, a multi-level pattern analysis was performed in R (multipatt function, package Indicspecies v.1.7.12, function “r.g,” α = 0.5). This analysis evaluates the relative frequency and abundance of taxa in each group and utilizes a permutation test to assign a test statistic or ‘indicator value’ to each ASV by group (40, 41) (Appendix 1—Script 3).

## RESULTS

This study included samples from three honey bee subspecies derived from six locations worldwide: African honey bees (*A. mellifera scutellata*, *n* = 82), European honey bees (*A. mellifera* sspp., *n* = 44), and Africanized honey bees (*A. mellifera scutellata* × sspp., *n* = 10) (Fig. 3; Table S1).

### Microbial composition and diversity by honey bee subspecies and provenance

**Fig 4:**
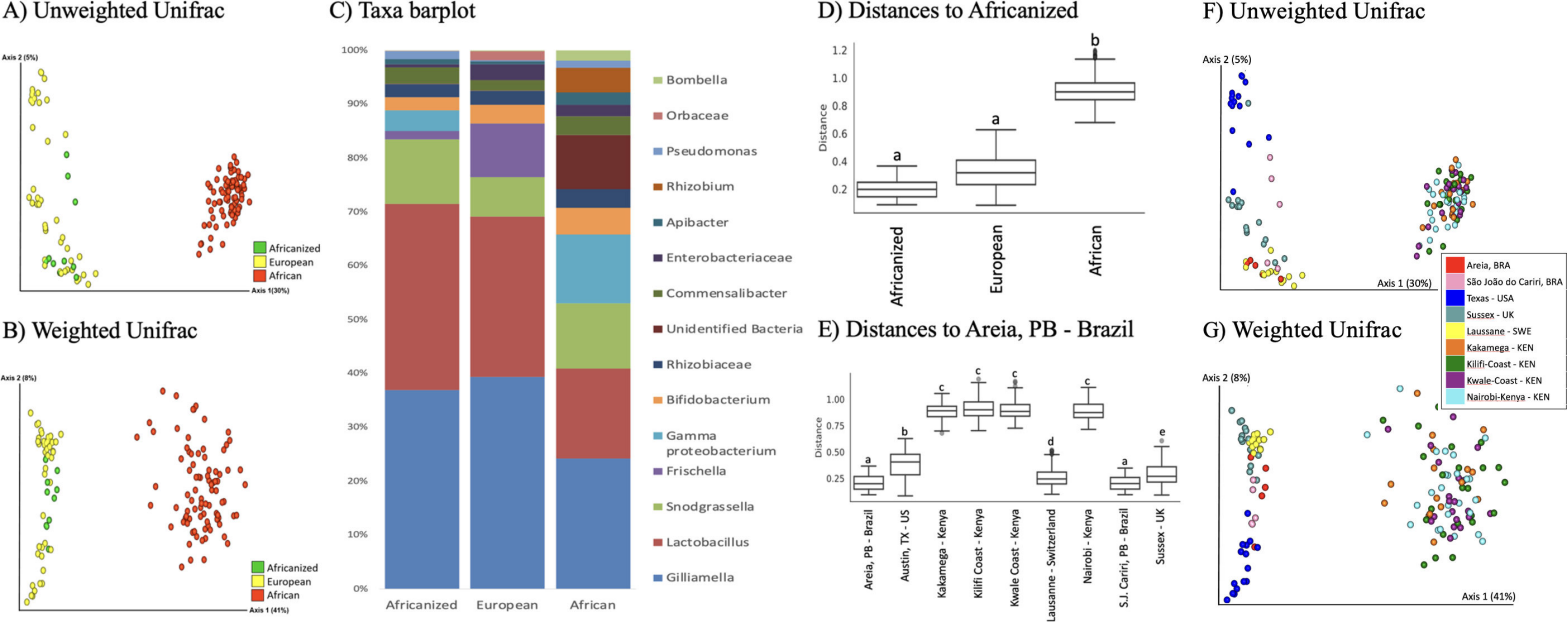
Gut microbial composition of European, African, and Africanized honey bees. Overall honey bee gut microbial composition differed significantly between all three honey bee subspecies based on (A) unweighted UniFrac and (B) weighted UniFrac metrics. (**C**) Taxa barplot of all microbial taxa found at >1% relative abundance and averaged across all samples from each honey bee subspecies. (**D**) Pairwise comparisons (weighted UniFrac) between honey bee subspecies (weighted UniFrac pairwise PERMANOVA: Africanized vs. European *P* = 0.136; European vs. African *P* = 0.001; African vs. Africanized *P* = 0.001). (**E**) Pairwise comparisons (weighted UniFrac) by honey bee provenance (weighted UniFrac PERMANOVA *P* = 0.001; see Table S3 for pairwise *P* values). Honey bee gut microbial composition also differed significantly between all sampling locations based on (F) unweighted Unifrac and (G) weighted Unifrac metrics (overall PERMANOVA: unweighted Unifrac *P* = 0.001, weighted Unifrac, *P* = 0.001).

**Fig 5:**
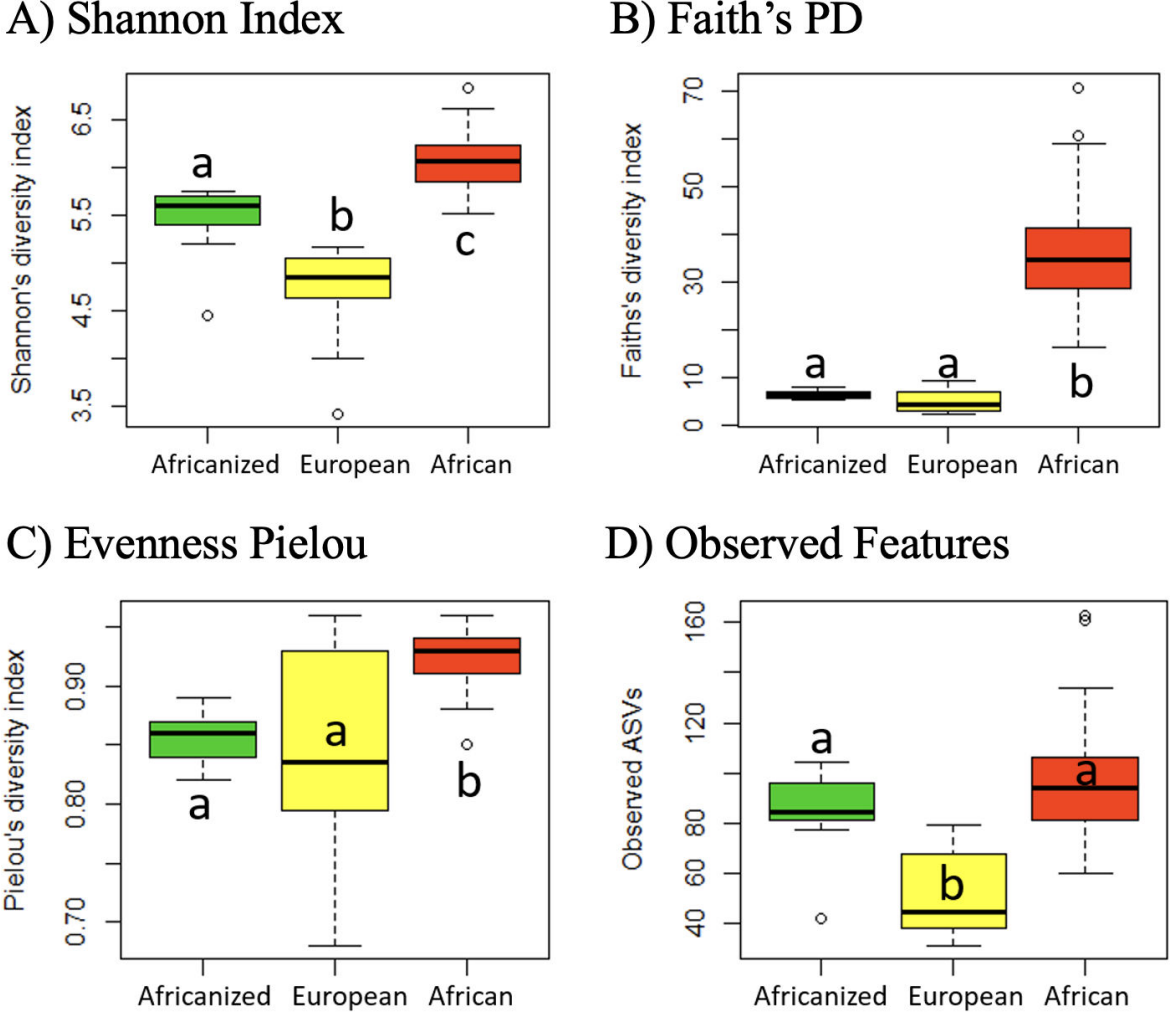
Gut microbial diversity of European, African, and Africanized honey bees. (**A**) Shannon index, (**B**) Faith’s PD, (**C**) Pielou’s evenness, and (**D**) observed features. Box plots show outliers, first and third quartiles (lower and upper edges), and highest, lowest, and median values (horizontal black dash). (Kruskal-Wallis: Shannon index *P* = 2.2e−16; Faith’s PD *P* = 2.2e−16; Pielou’s evenness *P* = 1.421e−08; observed features *P* = 2.2e−16). Significant differences (*P* ≤ 0.05 Tukey’s test) are denoted with different letters over each box plot.

We obtained a total of 919,168 raw reads across all samples, with an average of 6,565 reads per sample (range: 1,015 to 26,958 reads). Overall honey bee gut microbial composition differed significantly by subspecies (PERMANOVA: weighted Unifrac *R*^2^ = 0.4178, *P*-value = 0.001; unweighted Unifrac *R*^2^ = 0.3062, *P*-value = 0.001, Fig. 4; Fig. S1). However, based on pairwise comparisons (weighted UniFrac), European and Africanized honey bees did not differ significantly, while African bees differed significantly from both European and Africanized bees (Fig. 4D, weighted Unifrac pairwise PERMANOVAs: Africanized vs. European: *P* = 0.136; Africanized vs. African: *P* = 0.001, African vs. European: *P* = 0.001; unweighted Unifrac pairwise PERMANOVAs: Africanized vs. European *P* = 0.001; European vs. African: *P* = 0.001; African vs. Africanized *P* = 0.001). Microbial diversity also differed significantly between subspecies (Kruskal-Wallis: Shannon index *P* = 2.2e−16; Faith’s PD *P* = 2.2e−16; Pielou’s evenness *P* = 1.421e−08; observed features *P* = 2.2e−16, Fig. 5). Africanized honey bees exhibited a microbial diversity (Shannon diversity index) significantly greater than European bees but lesser than African bees. In terms of phylogenetic diversity (Faith’s PD) and evenness (Pileou’s index), Africanized honey bees were comparable to European bees, while in terms of microbial richness, Africanized honey bees were comparable to African bees.

In an effort to parse the effects of provenance (biogeography) versus host (subspecies), we then examined gut microbial composition by honey bee provenance. Gut microbial communities from all nine sampling locations around the world differed significantly, except the four locations in Kenya, compared to each other and the two locations in Brazil compared to each other (Fig. 4E, F and G; Fig. S2; Tables S2 and S3; weighted UniFrac PERMANOVA *P* = 0.001). Notably, the two locations in Brazil differed significantly on unweighted (PERMANOVA *P* = 0.013) but not weighted UniFrac (PERMANOVA *P* = 0.315) metrics, indicating that dominant gut microbial taxa and abundances were similar across locations, but the presence of rare taxa differed by sampling location (Tables S2 and S3).

### Core microbiota and differentially abundant microbial taxa

**Fig 6:**
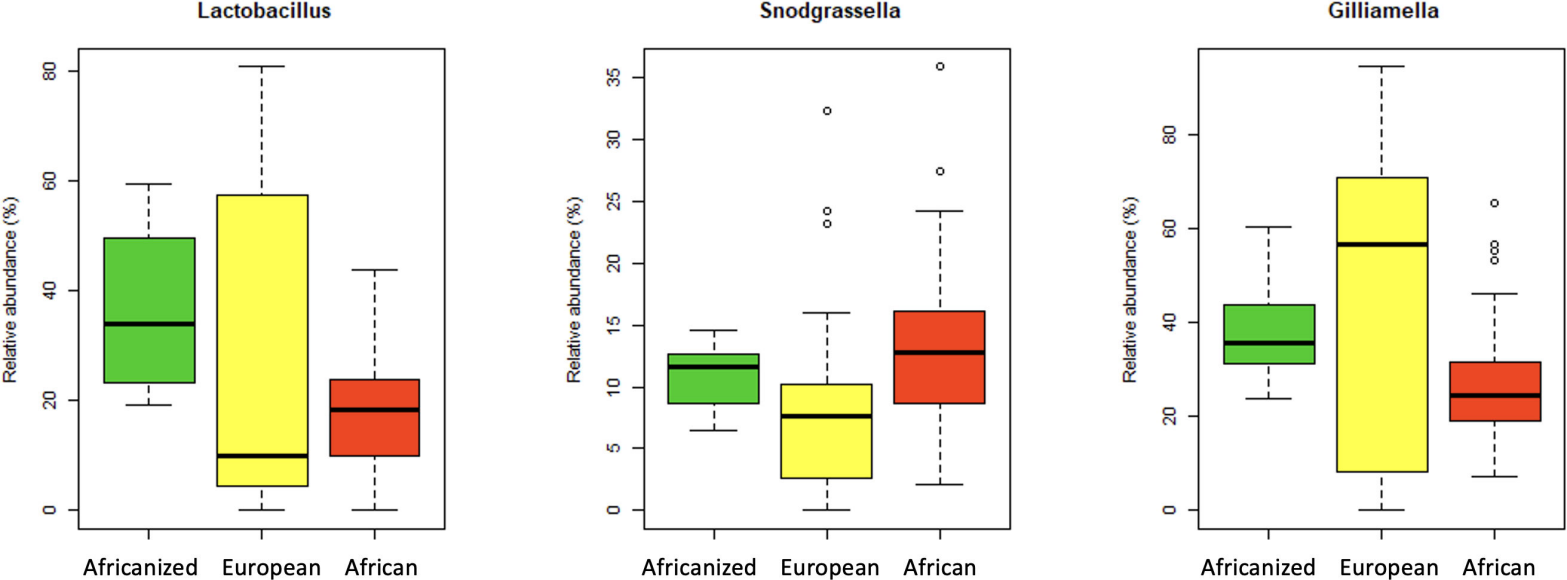
Relative abundances of core microbiota (genera) that were present in 90% of the samples across all subspecies. (A) Lactobacillus, (B) Snodgrassella, and (C) Gilliamella. Box plots show outliers, first and third quartiles, and highest, lowest, and median values. None of these taxa differed significantly in abundance by honey bee subspecies (Kruskal-Wallis all *P* > 0.4).

A core microbiota analysis identified three taxa (genera) that were present in 90% of the samples across all honey bee subspecies. These included *Lactobacillus*, *Snodgrassella*, and *Gilliamella* (Fig. 6). These taxa accounted for 15% of all genera in the data set. There were no significant differences in abundances of these taxa across subspecies (Kruskal-Wallis all *P* > 0.4).

**Fig 7:**
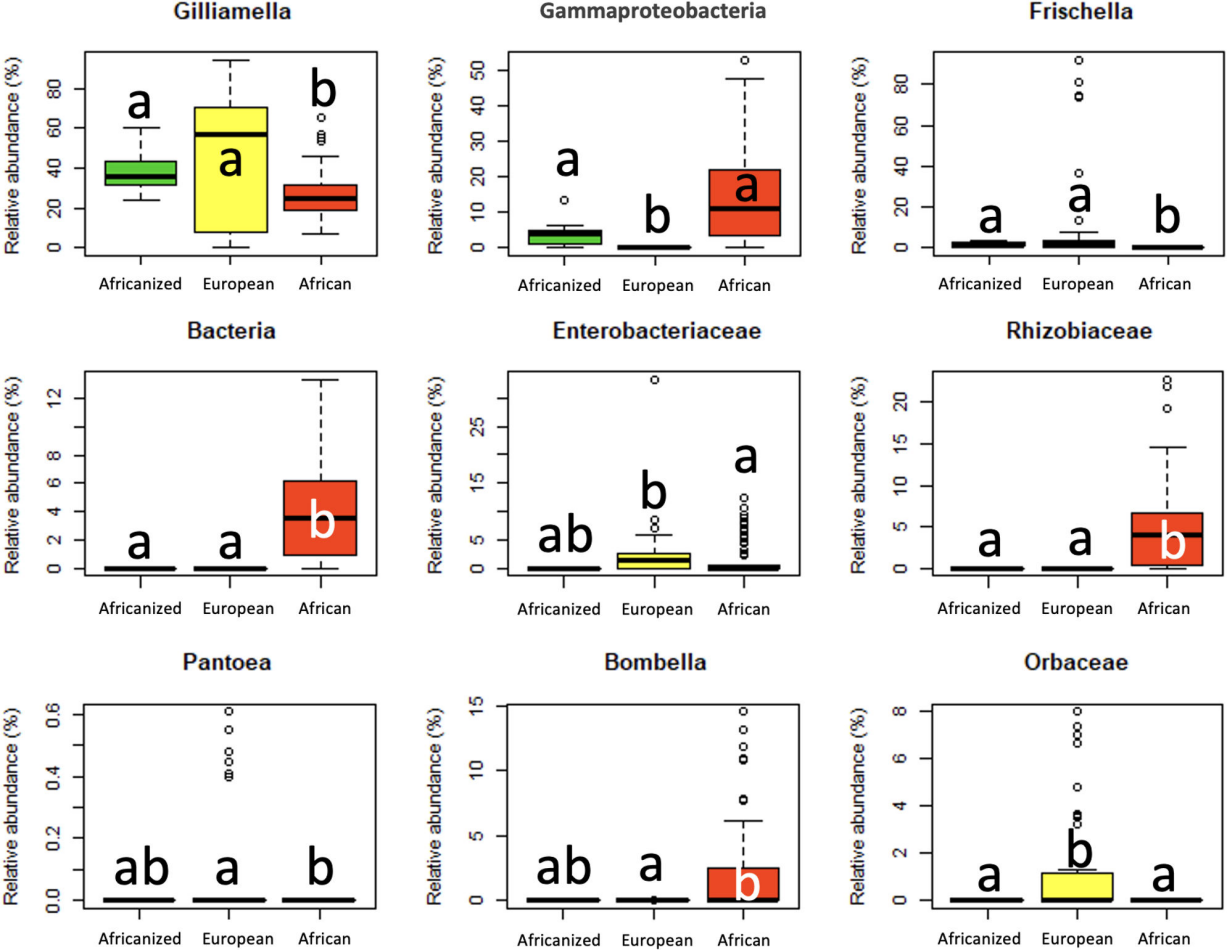
Relative abundances of differentially abundant genera (ANCOM) by honey bee subspecies. Box plot shows outliers, first and third quartiles, and highest, lowest, and median values. Statistical differences (Tukey’s test *P* ≤ 0.05) are denoted by different letters over each box plot.

Using an ANCOM, we then identified nine differentially abundant taxa (genera) by honey bee subspecies, including taxa in the families Orbaceae, Rhizobiaceae (Allorhizobium-Neorhizobium-Pararhizobium-Rhizobium) and Enterobacteriaceae, taxa from the genera *Frischella*, *Pantoea*, *Bombella*, *Gilliamella*, a taxa identified as a Gammaproteobacterium, and an unidentified bacteria (Fig. 7; Table S4). Africanized and African bees shared similar relative abundances of the Gammaproteobacterium and the Enterobacteriaceae, *Pantoea*, *Bombella*, and *Orbaceae* taxa. Africanized and European bees shared similar relative abundances of *Gilliamella*, *Fischella*, *Pantoea*, *Bombella*, the unidentified bacteria, and the Enterobacteriaceae and Rhizobiaceae taxa. African and European bees differed significantly in relative abundances of all these taxa.

### Microbial indicator species

To determine which microbial taxa distinguished each honey bee subspecies or subspecies pair, we performed an indicator species analysis (R package Indicspecies v.1.7.12). Taxa with an indicator value of >0.4 and *P* ≤ 0.05 were considered microbial indicator species. Indicator species analysis largely aligned with ANCOM results. Species within the Alphaproteobacteria class (e.g., *Bombella*) were key indicators of African bees, while Gammaproteobacteria species (e.g., *Pantoea*, *Frischella*) were indicators in European bees. An uncultured Gammaproteobacterium was shared between African and Africanized honey bees, while a *Gilliamella* species was shared between European and Africanized honey bees (Table S5).

## DISCUSSION

In this study, we compared gut microbiota of three honey bee subspecies (African, European, and Africanized) and found significant differences in microbial composition and diversity between subspecies and, to a lesser degree, by honey bee provenance. We hypothesized that Africanized honey bees, which are a hybrid between African and European honey bees, would have gut microbial communities that shared features with both African and European bees. Our results supported this hypothesis. We further hypothesized that Africanized honey bee gut microbiota would be most similar to African honey bee gut microbiota, as these two subspecies are most closely related phylogenetically. However, our results did not support this hypothesis: instead, we found that Africanized and European honey bees had much more similar gut microbial communities than African and Africanized bees despite being more phylogenetically distant (less genetically related) based on host (honey bee) genes. We explore potential explanations for this finding below.

### Africanized and European honey bees exhibited highly similar gut microbiota despite phylogenetic distance between hosts

Africanized and European honey bees exhibited similar overall gut microbial composition and dominant taxa (weighted UniFrac), similar phylogenetic diversity (Faith’s PD) and evenness (Pileou’s), and similar abundances of many common honey bee taxa (e.g., *Gilliamella*, *Frichella*, *Bombella*, and taxa in the Rhizobiaceae family), with a *Gilliamella* species notably serving as an “indicator species” found at ~35–60% relative abundance in European and Africanized bees and only up to ~25% relative abundance in African bees. We posit that the similarity between Africanized and European bee gut microbiota may be due to honey bee hive dynamics and sociality in shaping microbiota transmission and acquisition. Specifically, honey bees acquire gut microbiota through contact with nurse bees, fecal material, and hive components (comb, honey, bee bread) (21, 42). Gram-positive bacteria, such as *Lactobacillus* or *Bifidobacteria*, are typically acquired through contact with hive surfaces and nurse bees, including via trophallaxis (nutrient exchange by mouth from nurse to newly eclosed adult bees) (42–44). Acquisition of gram-negative bacteria, such as *Gilliamella*, *Frischella*, and *Snodgrassella*, relies on contact with fresh fecal material in the hive or contact with nurse bees (not including trophallaxis) (42). This social and environmental gut microbial transmission/acquisition process ultimately leads to stable coadapted gut microbial communities across generations in adult honey bees (29, 42, 45–48).

After African queens were accidentally released in Brazil, they eventually established and crossbred with European bees. Although African queens may have maintained their own distinct gut microbiota, in some cases, they likely ended up in hives with European nurse bees and European bee hive materials. In these cases, even though the newly emerging Africanized bees were genetically more closely related to the African queen, these hybrids were acquiring gut microbiota via contact with European nurse bees, fecal material, and hive components. This idea is supported by Tarpy et al.’s study (49) demonstrating that the queen gut microbiome is distinct from and not transmitted to offspring within a hive, and that there are only modest changes to worker bee microbiota (in terms of relative abundances) before and after queen replacement. Ultimately, the European bee gut microbiota may have also provided fitness benefits to the Africanized hybrids, as European bees had been present in and adapting to the Brazilian climate and environment for over 100 years before African bees were introduced.

### Africanized and African honey bees maintained few similarities in gut microbiota despite phylogenetic closeness between hosts

African and Africanized honey bees shared fewer similarities in gut microbiota as compared to European and Africanized bees. African honey bee gut microbiota was highly distinct from Africanized bees and generally more diverse (Shannon index, Faith’s PD, Pileou’s evenness). This could be due to minimal transmission of gut microbiota from African queens to Africanized hybrids or to differing diets, dietary diversity, environments, or environmental pressures faced by African bees as compared to European and Africanized bees (29, 50, 51). A few taxa, including Gammaproteobacteria and a taxon in the Orbaceae family, were found at similar abundances in African and Africanized bees. Gammaproteobacteria abundances have been reported to increase in response to stressors, including parasite and pesticide exposures (52). It is feasible that honey bees in Africa and Brazil faced some similar stressors, and that increased Gammaproteobacteria abundances represent a response to these stressors. It is also possible that Gammaproteobacteria provide fitness benefits to honey bees in both Africa and Brazil (e.g., pathogen resistance, rapid colony growth, adaptability to new environments) (12, 53). Alternately, these taxa may be particularly well suited to social transmission (e.g., from queen to workers to offspring) (49) and environmental acquisition (e.g., ecological constraints) or have microbiome-host genetic associations linked to African honey bee genes.

### Core microbiota across honey bee subspecies

*Lactobacillus*, *Snodgrassella*, and *Gilliamella* were identified as core microbiota present in 90% of the samples and found across all honey bee subspecies. Other studies report similar findings in terms of core microbiota (29, 54). Notably, *Bifidobacterium* was also considered a core microbe in honey bees (29) but was only present in 80% of the samples in our study and therefore did not meet our “core” threshold. Fourteen (out of 82) samples from African bees lacked detectable *Bifidobacterium*, which may have been an artifact of sample preservation, extraction, library preparation, or sequencing, as *Bifidobacterium* is generally found at lower abundances in the honey bee gut (45).

### Gut microbiota and honey bee provenance

We observed the most pronounced differences in honey bee gut microbiota by host phylogeny (subspecies), but we also observed differences in gut microbiota based on honey bee provenance. Provenance is linked with environment and diet, and multiple studies have reported differences in gut microbiota based on provenance in various animal species, including honey bees (18, 32, 45, 55). Notably, as our study employed publicly available data from other studies, there were differences in sample preservation, extraction, library preparation, and sequencing methods that potentially confounded these analyses (Table S1). This was particularly true across European bee samples/studies. However, all African honey bee samples (from four sampling locations across Kenya, central to coast) were collected, extracted, and sequenced in the same manner, and when these technical variables were controlled for, no differences were detected in gut microbiota by sampling location in African honey bees. These results support the original findings of Tola et al.’s study (31). Interestingly, the honey bee samples from the two locations in Brazil were also preserved, extracted, and sequenced in the same way but *did* differ significantly in rare taxa (unweighted Unifrac), albeit not in dominant/abundant taxa (weighted Unifrac) (Tables S2 and S3). Taken together, this suggests that provenance does impact honey bee gut microbiota, but that host phylogeny may play a larger role in shaping bee gut microbial communities.

### Conclusion

Our study provides key insights into potential factors that have shaped Africanized honey bee gut microbiota co-evolution. Namely, our results suggest that the introduction of African queens to European hives and the social/environmental transmission of gut microbiota within these hives lead to an Africanized honey bee hybrid that is phylogenetically more African while hosting a gut microbiota that is more European. Provenance can also play a role in shaping honey bee gut microbiota, although to a lesser degree. Deeper examination of microbial phylogenies between honey bee subspecies and evaluation of honey bee fitness across environments with differing microbial strains could further elucidate the mechanisms underlying these patterns. Examining the complex relationships between honey bees and their gut microbiota is critical for understanding honey bee evolution, for conservation efforts and the preservation of host and microbial genetic diversity in honey bee populations, for beekeeping practices, and ultimately for protecting the health and sustainability of these vital pollinators.

## Data Availability

The data sets utilized in this study are all publicly available via online repositories. See Table S1 for a list of all National Center for Biotechnology Information Bioproject numbers and Zenodo links. Supplemental figures and tables are accessible at FigShare: https://doi.org/10.6084/m9.figshare.28319087.v2. Appendix 1 (QIIME2 and R scripts) is accessible at this FigShare link: https://doi.org/10.6084/m9.figshare.28319051.v1.
